# Silent Witnesses: Exploring the Impact of Cone Beam Computed Tomography on Forensic Investigations

**DOI:** 10.7759/cureus.96401

**Published:** 2025-11-09

**Authors:** Prashanthi Reddy, Ganiga Channaiah Shivakumar, Vanaja Reddy, Neha Mehrotra, Pratiksha Kumar, Anuradha Agrawal, Vishnu Desai, Santosh Kumar, Rahnuma Ahmad, Mainul Haque

**Affiliations:** 1 Oral Medicine, Government College of Dentistry, Indore, IND; 2 Oral Medicine and Radiology, People's College of Dental Sciences and Research Centre, Bhopal, IND; 3 Oral Medicine and Radiology, Modern Dental College and Research Centre, Indore, IND; 4 Periodontics, Index Institute of Dental Sciences, Indore, IND; 5 Oral Pathology and Microbiology, Government College of Dentistry, Indore, IND; 6 Dentistry, Government College of Dentistry, Indore, IND; 7 Periodontology and Implantology, Karnavati School of Dentistry, Karnavati University, Gandhinagar, IND; 8 Physiology, Medical College for Women and Hospital, Dhaka, BGD; 9 Pharmacology and Therapeutics, National Defence University of Malaysia, Kuala Lumpur, MYS; 10 Scientific Committee, Global Alliance for Infections in Surgery, Macerata, ITA; 11 Research, Karnavati School of Dentistry, Karnavati University, Gandhinagar, IND; 12 Pharmacology and Therapeutics, Eastern Medical College and Hospital, Cumilla, BGD

**Keywords:** age estimation, bite marks, cone beam computed tomography, cranial trauma, facial reconstruction, forensic identification, forensic odontology, radiology in forensics, sex determination, teeth

## Abstract

Cone beam computed tomography (CBCT) has transformed forensic odontology by contributing high-resolution, low-dose, three-dimensional imaging capabilities. This review emphasizes the applications of CBCT in forensic investigations, including personal identification, age estimation, sex determination, bite mark analysis, ancestry estimation, facial reconstruction, and assessment of cranial trauma. CBCT's versatility, precision, and noninvasive nature make it a valuable tool in both postmortem and antemortem investigations. This article also discusses the current limitations of CBCT and outlines future research perspectives in forensic roentgenology.

## Introduction and background

Forensic dentistry, also known as forensic odontology, is a vital field within forensic science that utilizes dental know-how to identify both deceased and living individuals. As stated by the American Academy of Forensic Sciences, this discipline plays a pivotal role in various scenarios, such as mass disasters, detection of injuries, and cases of abuse, ultimately aiding in the identification and protection of victims [1]. The purpose of the identification process is to compare the characteristics of individuals before and after their disappearance to establish their identity [2,3]. Teeth are often utilized in forensic investigations because they remain well-maintained over time, irrespective of the somatic condition of the deceased body [4]. Because teeth hold up well as time goes on, independent of the condition of the body, they are frequently used in forensic examinations [5-7]. Teeth are less impaired by ecological, endocrine, or glandular secretions, as well as innate aspects, than the remaining parts of the human body, and they are more resilient to exogenous compounds and physical forces or agents, in conjunction with long-term dietary nutritional deficiencies [8-10]. Some of the features that surround the teeth, such as the sinuses or facial bones, are also fascinating [11]. Over time, several methods have been developed to investigate these structures. In specific religious, cultural, or scientific contexts, some of them were extremely intrusive and damaging, posing moral dilemmas for both the living and the dead [12-14].

A 2D detector system is at the center of a cone-shaped X-ray beam used in cone beam computed tomography (CBCT). Based on a sequence of 2D frames, these components revolve around the body part being inspected to create a 3D representation [15-17]. CBCT is already widely used for head and neck imaging for diagnostic purposes in almost all fields of dentistry, including maxillofacial, periodontics, orthodontics, implantology, and endodontic surgery [15,17]. CBCT is a nondetrimental and nonintrusive technique. It provides better voxel resolution, quick scan times, precise and thorough anatomical information, and good image quality [18-20]. Using a sizable voxel and a small field of vision can reduce the radiation. It enables precise reconstruction free from geometric distortion and a multidimensional scan (sagittal, axial, and coronal planes) [21,22].

Forensic identification is imperative for both legitimate and reconciliation intents, particularly during the post-mortem (PM) phase. However, if there is a mass killing resulting from terrorism or a natural disaster, this process becomes extremely complicated. The antemortem (AM) and PM records are compared to identify the individual. As one of the human body's toughest tissues, teeth can be identified through forensic odontology. Radiology is a crucial component of identification in forensic odontology.

In contrast to the utilization of AM and PM information, dental imaging creates a photographic image of the dental framework and composition, which is compared to written notes, dental diagrams, and proceedings or records to facilitate easier identification of victims [23,24]. The use of suitable techniques facilitates the distinguishing procedure in forensic radiology. Technicians or professionals with a variety of specialties and appropriate methodologies can build the procedure [25]. If AM records are adequate, the use of X-ray imaging in pinpointing can be evaluated. Radiography can be used to study a variety of morphological and pathological changes, including the morphology of the crown and dental roots. By matching the AM and PM data from the radiograph, the identification was completed. A diastema, bone pathology, alveolar bone resorption from periodontal disease, tooth silhouette and root, number of teeth or deformities in the number of teeth, tooth malposition, abrasion conditions, coronal fractures, prior endodontic therapeutic interventions, dentures, presence of implants, interradicular and post-treatment changes within the crown, and extraction marks are some instances of dental framework details that can be used for identification [26]. The identification process is thought to be substantially aided by the benefits of CBCT digital radiography, primarily due to its speed, simplicity of viewing on a computer monitor, and potential to manipulate image parameters (e.g., contrast, magnification, and brightness), as well as the repurposing of digital images compared to conventional film radiographs, which permits more thorough analysis and comparison across different contexts [27].

Dental professionals' interest in dentomaxillofacial CBCT scanners has skyrocketed since their introduction in the late 1990s. Its modest dosage and comparatively inexpensive cost are its clear advantages. In contrast to CT, CBCT utilizes a single X-ray source that emits a cone-shaped beam of radiation, rather than a fan-shaped beam of radiation. A single, reasonably priced, flat-panel or image-intensifier radiation detector is used in CBCT. A rotating platform, with the X-ray source and detector mounted on it, is used for CBCT imaging. Multiple, consecutive, multiplanar images are created as the X-ray source and detector revolve around the object; these images are then scientifically renovated into volumetric facts and statistics [28]. It possesses numerous benefits for predeceased and posthumous forensic-pathological tomography, including high resolution for skeletal imaging, affordability, mobility, and ease of use. CBCT may be highly beneficial in specific forensic procedures [29]. It provides a non-surgical method for estimating age, which is crucial for forensic dentistry [30]. As people age, the pulpo-dentinal complex - which includes the tooth pulp, cementum, and dentin - shows both physiological and pathological alterations [31]. Forensic identification has primarily relied on radiograms of the face, long bones, teeth, and skull. Facial radiographical images are superior among them, primarily due to the existence of several geometrical characteristics that enable accurate superimposition into an identity [32].

Bishop Oscar Romero quoted, "Let your voice be a bridge for the unheard" [33]. Forensic science, which establishes the identification of the deceased and unidentified by physical, functional, mental, regular, or abnormal traits, is therefore crucial for people without a voice. A methodical comparison of the deceased person's AM and PM data is used in the forensic identification process. One of the human body's strongest structures is the maxillofacial bones and teeth, which can withstand taphonomic processes and destruction for a very long time, despite changes in temperature and chemical composition. They are the strongest bodily structures because they can withstand taphonomic processes and destruction for a very long time, even when exposed to changes in temperature and chemical composition [34]. Radiology is a necessary instrument for forensic science. In the event of a forensic inquiry, it helps compare AM and PM records, in addition to assisting with the examination of anatomical structures for personal identity, biologic age estimation, evaluation of physical violence or torture, and verification of the armament of grievous bodily harm. Prof. Arthur Schuster demonstrated the presence of lead bullets in a victim's head using radiography for the first time in forensics in 1896 [35]. Three-dimensional imaging in forensic identification is necessary to overcome the drawbacks of 2D radiography, such as dimensional variances in images and different vertical and horizontal angulations [36]. Sir Godfrey Hounsfield, in 1971, began the era of 3D imaging with X-ray CT. Hounsfield's archetype scanner technique was first utilized for clinical CT scans at Atkinson Morley Hospital in Wimbledon, London, UK, in 1971.

Furthermore, it has been reported that Dr. Paul Lauterbur first hypothesized the concept of MRI. Dr. Lauterbur published the principle for MRI in 1973 and received the Nobel Prize in 2003. The information from AM CT scans can be used to create PM facsimile imaging. Robles RA initially constructed CBCT scanners in 1982 for angiography; however, they have since evolved into a crucial 3D imaging technology, particularly in dentistry [37-40].

Problem statement

Traditional forensic identification techniques often face limitations in resolution, accessibility, and invasiveness. There is a pressing need for advanced imaging technologies that can provide detailed anatomical data with minimal damage to human remains. CBCT offers a viable solution, but it requires a comprehensive understanding of its applications, benefits, and challenges in forensic science.

Objectives of this narrative review

**Figure 1:**
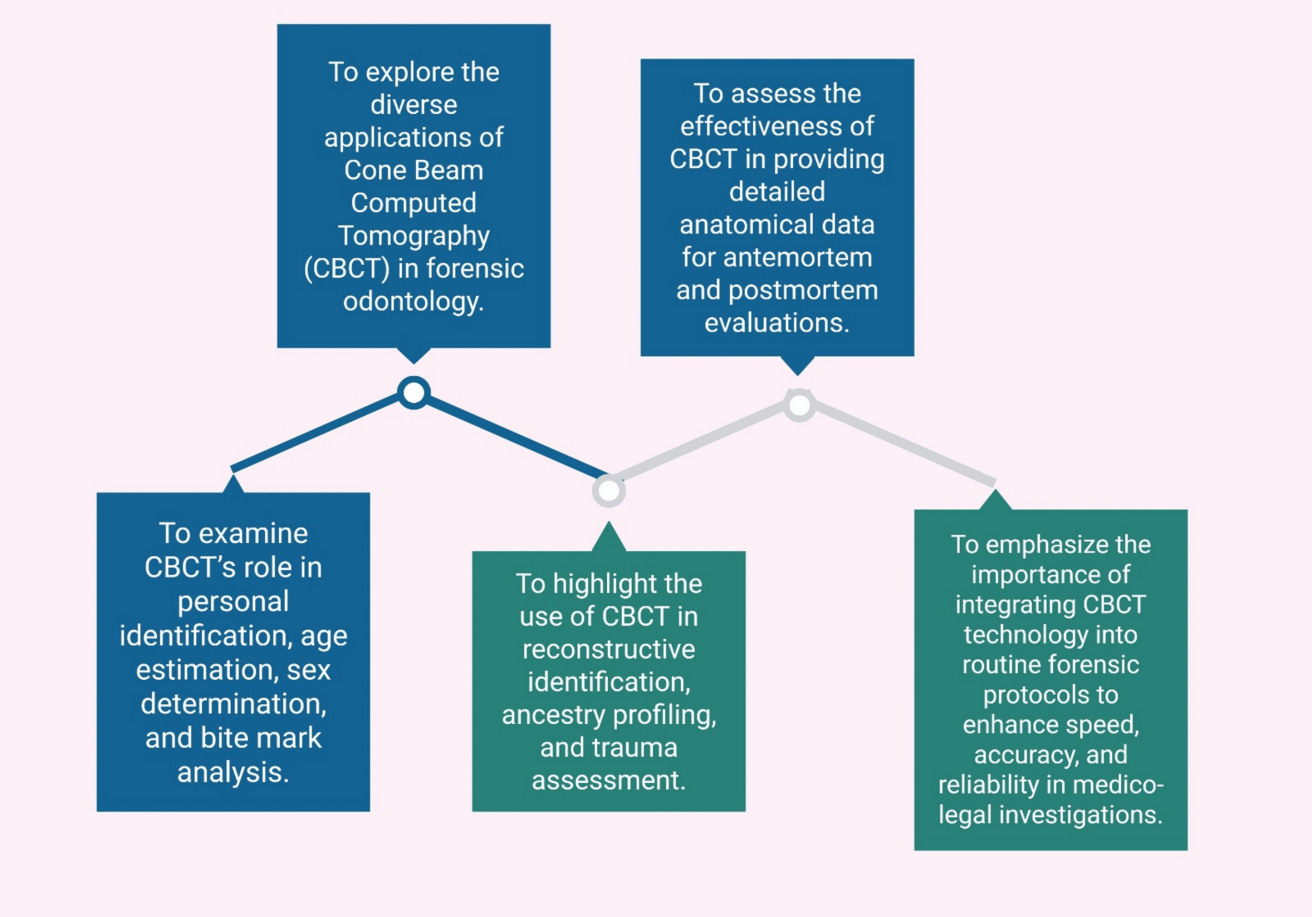
A flow chart illustrating the objectives of the study Note: This figure was drawn using the premium version of BioRender (https://BioRender.com/hz6ldv0) [41], accessed June 27th, 2025, with an agreement license number UW28FTVPZF. Illustration Credit: Vishnu Desai

The primary objective of this review is to investigate the diverse purposes of CBCT in the field of forensic odontology. This includes examining its role in personal identification, age and sex estimation, and bite mark analysis. This narrative review set out to assess the effectiveness of CBCT in providing detailed anatomical information that supports both AM and PM evaluations. Additionally, it seeks to highlight CBCT's potential in reconstructive identification, trauma assessment, and ancestry estimation. By analyzing current literature, this article also aims to identify the limitations of CBCT in forensic settings and propose directions for future research and clinical integration (Figure 1).

## Review

Materials and methods

Study Design

This narrative review assessed the diverse applications of CBCT in forensic odontology, aiming to identify its limitations in forensic settings and to propose directions for future research and clinical integration.

Eligibility Criteria

Inclusion criteria: Peer-reviewed articles published between 2000 and 2024, written in English, and focusing on CBCT applications in forensic contexts.

Exclusion criteria: Case reports, book chapters, letters, conference abstracts, and personal opinions; non-English articles; articles unrelated to forensic applications of CBCT; abstracts without full-text availability.

Information Sources and Search Strategy

A comprehensive literature search was conducted in the following databases: PubMed, Scopus, ScienceDirect, and Google Scholar, covering references published between 2000 and 2024. Search terms included: "CBCT in forensic odontology," "Cone Beam Computed Tomography forensic," "CBCT age estimation," and "3D imaging forensic dentistry."

Study Selection Process

A database search revealed 1,250 titles. In the initial screening phase, 252 duplicate articles were excluded. Titles and abstracts were then assessed, resulting in the exclusion of one non-English study, 620 studies not related to forensic CBCT, 121 abstracts only (no full text available), and subsequently, 742 additional papers were omitted because they did not meet the eligibility criteria when checked for full text.

Eligibility was assessed for studies published between 2000 and 2024, focusing on the forensic application of CBCT and on those with full-text availability. Two hundred fifty-six articles advanced to eligibility assessment. Then, case reports, personal opinions, book chapters, letters, and conference abstracts (100) were further removed.

Data Extraction

After rigorous review, 156 manuscripts were included in the study. Information on CBCT forensic applications, benefits, limitations, and outcomes was extracted and synthesized thematically.

Quality Assurance

**Figure 2:**
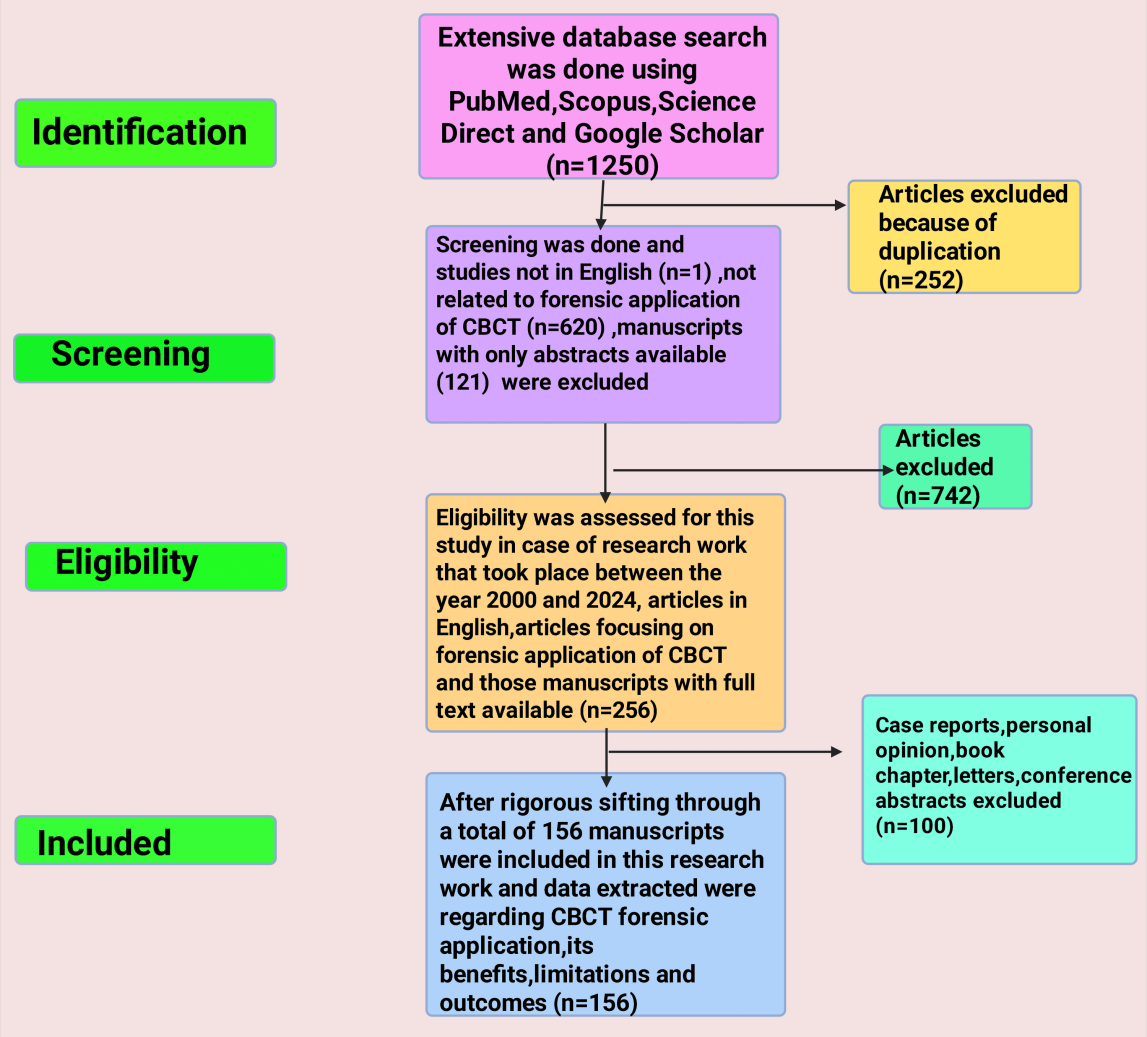
Displaying the flow chart for the selection process of the study Note: This figure was created using the premium version of BioRender (https://biorender.com/) [41], accessed on September 23rd, 2025, with license number ZT28SG5WUF. Illustration Credit: Rahnuma Ahmad

At least one reviewer collected information, with another reviewer verifying the accuracy of the synthesis process (Figure 2).

Review of literature

Revolutionizing Diagnostics: The Role of CBCT

All areas of dentistry, including orthodontics, endodontics, oral surgery/pathology, periodontics, and implant treatment planning, are making extensive use of the ground-breaking finding known as CBCT. It is a medical imaging method that uses a cone-shaped X-ray beam and a flat-panel detector to capture a rotational (180°-360°) scan of an object. The multiple images attained during the rotation are then prepared by mathematical reconstruction or geometric constructions, with the Feldkamp algorithm being one such method, to create a 3D volumetric dataset for visualization and analysis [42]. The CBCT data can also be introduced through negotiator image-enriching programs, e.g., “3-Matics (Materialize NV, Leuven, Belgium) and Mimics (Materialize NV, Leuven, Belgium) for 3D volumetric analysis” [6,43,44]. These programs have been utilized in various forensic procedures and analyses [45]. The benefits of CBCT include reduced radiation (approximately 96% less than traditional CT), improved mobility, shorter image reconstruction times, and fewer severe metallic artifacts [46].

CBCT analysis of radiologic representation or depiction is a technique that is increasingly being used in PM photographs for forensic investigations, including age and sex examination. Virtopsy (virtual autopsy) is an examination procedure - a non-invasive PM examination - for locating foreign matter or contaminants. The superiority of CBCT over projection images lies in its ability to provide a more thorough depiction of skeletal or ossified tissue, including structures and pathways that appear clear regardless of the projection angle. Although most of these analyses are conducted in labs or morgues, field analysis is increasingly using them. In recent years, CBCT has gained popularity for PM exams due to its lower cost. However, since CBCT is not the most practical substitute that can be utilized in all forensic cases, the drawback of therapeutic CBCT is the inconvenience in reading its use [25,47].

Bite Marks as Evidence: From Impression to Identification

Tooth impressions can now be transformed into more reliable substantiation due to the development of CBCT. Wu et al. (2013) created comparison overlays automatically by comparing the overlays of doubtful bite marks on dental casts with CBCT imageries and plate visualizing [48]. In comparison to the traditional method, the blind identification of two types of bite mark data demonstrated that CBCT, when used for bite mark identification, had a very high specificity, making it an accurate and valuable tool for this purpose. Marques et al. (2013) [49] and other studies have demonstrated that it is feasible to conduct a metric analysis of bite marks in suitably thick foods, using CBCT to examine tooth wound inscriptions in edibles that would be discovered in a forensic case investigation outfit [49,50].

Evidence of bite marks on victims and objects may be crucial in criminal investigations. The bite mark is a 3D phenomenon, as is the dentition that causes it [51,52]. Information loss and distortion are implied by the bidimensional registration of 3D structures [53]. A well-established method for comparing bite marks involves measuring and comparing the size, shape, and pattern of teeth to similar marks left on the object. When one person bites, three 3D elements are involved: the depth of penetration, the shape of the biting teeth, and the curvature of the object. Because the object is deforming to fit the shape of the teeth, the damage is a 3D event. The mark is also 3D if the bite force is sufficient to make an impression in the object. CBCT, explicitly devised for imaging dental structures, requires less expensive equipment, has lower radiation doses (around <15-fold) than traditional CT scans, and utilizes relatively small equipment. The pictures are acquired as DICOM (Digital Imaging and Communications in Medicine) files, which can be examined using various software programs [54-56].

Analysis of bite marks is crucial to forensic identification and inquiry. Bitemarks are indications made by teeth, either from one's own bites or those of others. Everyone has exclusive bite marks and forms, which are typically observed in relation to homicides, child abuse, and sexual assault. Bitemarks are frequently discovered on the bodies of deceased and living individuals, as well as on items close to the crime scene. To make identification easier, victims often observe bite marks on the female genitalia, breasts, waist, arms, legs, neck, and face, as well as on items like apples, cheese, and chocolate [57]. Using 3D CBCT for reconstructions, Giri et al. (2019) examined bite marks on apples to determine the students' bite patterns [57]. When it came to identifying bite patterns, CBCT radiography performed a substantially more detailed analysis than standard radiographic analysis, using CBCT to analyze the relative density of bite marks on various culinary products, e.g., apples, cheese, pizza, tarts, chocolate, and gum. Marques et al. (2013) further corroborate the earlier findings [49]. When comparing CBCT images to the previously acquired dental model, the analysis can be exact, particularly when it comes to the bitemark pattern [57,58].

Forensic Age Estimation: A Multidisciplinary Perspective

In a forensic setting, age estimation is a crucial component of biological profile estimation [4,5,7,13,54,59-62]. Growth-related, geomorphological, chemical, and organic transformations in teeth are the basis for several dental age assessment techniques; CBCT enables a 3D, nonintrusive analysis [9,63]. The study of pulp narrowing is one of the most popular techniques for estimating age. Following tooth development and eruption, apical closure occurs, marking the beginning of dental function. Secondary dentin then starts to deposit [64,65]. Several studies have employed a linear regression model, even though this event occurs throughout life and causes a steady decrease in pulp volume, which exhibits a non-linear relationship with chronological age [66-69]. In the absence of advanced 3D imaging, the pulp, a 3D structure, was previously investigated using 2D radiography, which introduced distortion problems. Since secondary dentin deposition (SDD) is not uniform throughout the pulp, volumetric quantification is preferable over surface or linear measures when analyzing pulp narrowing for age calculation. These days, CBCT has proven to be an accurate method [70].

The purpose of age determination of human remains is to help establish an individual's age in the event of a lawsuit or when constructing a biologic précis of a departed person. A volumetric study of dental geographies in radiograms, such as narrowing or obliteration of the pulp chamber/canal, that results in secondary dentin production, helps determine an individual's age because SDD is closely related to age [71]. In 1925, Bodecker provided the first explanation of this association [72]. The following three steps are used to classify it: (A) newborn infant, immediately after birth, and antenatal: this stage is determined by the maxilla and mandible of the fetus, the way tooth germs form, the degree of crown completeness, and the early mineralization of primary teeth during intrauterine life; (B) juveniles and youngsters: this stage is determined by the presence of open apices in teeth, the degree of crown completion, the eruption time of a tooth in the oral cavity, the appearance of tooth germs, the earliest discernible trace of tooth mineralization, the magnitude of root accomplishment of erupted and/or unerupted teeth, and the range of root decay or structural damage of milk or primary teeth; (C) adult: this covers the development of the third molar and the measurement of the pulpal tissue volume of teeth [71].

Estimating dental age is crucial for clinical and forensic work. Age information is relevant to both criminal liability and marriage law in the field of forensic science, and it pertains to both living and deceased people. The most effective way to prevent destruction is to use teeth, which are also helpful for forensic identification of skeletal or decayed remains [73]. One of the dental structures that changes with age is the pulp-dentinal complex, which primarily causes the pulp chamber volume to decrease due to the ongoing accumulation of secondary dentin. While the endodontic space and root canals are broad in immature teeth that have just finished growing, they gradually contract with time and frequently reach near total obliteration due to SDD. Destructive methods, which employ tooth sections, and non-destructive methods, which utilize panoramic and periapical radiography, form the foundation of research to measure the quantity of secondary dentin in adults [74]. The alteration of dental hard tissue mass brought on by aging can be precisely and accurately measured with the CBCT. Conceivably, due to the presence of secondary dentin, potential sites may provide an inaccurate sense of the degree of this process; examining the pulp cavity and tooth volume is more accurate than computing areas [74].

Dental and Skeletal Indicators of Sex

There are several ways to assess sex, which is a crucial component of the biological profile. Although they highlighted its limitations in forensic practice (such as expense, logistical challenges, and the availability of biochemical products), Capitaneanu et al. (2017) found that biochemical analysis was the most accurate method for estimating odontological sex [75]. One helpful tool for CBCT study is the tooth itself. Male teeth are often bigger in diameter [76,77]. Manhaes-Caldas et al. (2019) analyzed the crown itself, including the enamel and dentin, and found that a mean accuracy of 83.7% could be achieved by combining the examination of the upper and lower canines. Because chromosome X solely affects amelogenesis, while chromosome Y promotes both amelogenesis and dentinogenesis, this dimorphism in crowns can be explained. This could explain why men's crowns are larger than women's [78]. Mandibular canines are said to have the highest sexual dimorphism among teeth. They are commonly employed because of their greater resilience to severe traumas or periodontal disease [78].

Determining a person's sex is crucial to estimating their biological profile. The skull, long bones, and pelvis are the bones that are typically used to determine an individual's sex. One hundred percent of skeletons can accurately establish a human's sex, 98% of skulls and pelvises can do so, 95% of pelvises or pelvis and long bones can do so, 90%-95% of skulls and long bones can do so, and 80%-90% of long bones alone can do so [79]. An individual's sex can be determined by anthropometric measures of several craniofacial features on CBCT scans, including the mastoid process, sinuses, nasal septum, foramen magnum, and mandible [33]. The size and form of the mandible show sexual dimorphism, which can be utilized to estimate sex highly accurately [80,81]. Due to factors including sex, diet, and physical activity, the bones of the female bone framework are smaller and thinner than those of males. The manifestation of mandibular dimorphism is influenced by the relative development (size, strength, and angulation) of the masticatory muscles, as males and females exhibit different masticatory forces [82]. Many measurements of the mandible, including bicondylar breadth, gonion-gnathion length, gonial angle, bigonial breadth, and ramus length and breadth, can be used with CBCT to determine gender [83]. Using a variety of additional anatomical landmarks, including orbital openings, volumetric evaluation of dental crowns, the morphology of the articular distinction and mandibular crater, mandibular centerline neurovascular canal configurations, and cervical vertebral contours, CBCT has proven to be a helpful tool for determining gender and identifying individuals [15,78,84,85].

Anthropometric assessments of mandibular images from CBCT can be used to evaluate sexual dimorphism [3]. The sexual dimorphism analysis includes six metrics: ramus and gonion-gnathion extent, least ramus extensiveness, gonial bend, bicondylar, and bigonial span [83,86]. Three of the six mandibular measurements have mean values that are noticeably greater in males than in females. These are minimal bicondylar breadth, gonial angle, and ramus breadth [87-90]. To determine which mandibular measurements provide the most insight into variations between boys and girls, a study was conducted among white South Africans [91]. The most significant discriminating factor was shown to be bigonial breadth, which had an average mandibular accuracy of 82% [92,93]. Anthropometric assessments were employed by Gamba Tde et al. (2016) on mandibular images acquired using CBCT. A sample of 159 CT scans from a Brazilian population (74 men and 85 women), between the ages of 18 and 60, comprised the study. Five reviewers examined the CBCT photos [3]. To analyze sexual dimorphism, six measurements were gathered: ramus length, gonion-gnathion length, minimum ramus breadth, gonial angle, bicondylar breadth, and bigonial breadth. The rate of accurate sex classification using these four factors was 95.1% [3].

Facial Reconstruction

The goal of face reconstruction is to recreate missing or unidentified facial features on an unnamed person's skull [94,95]. The goal is for family members and relatives to recognize it, or, at the very least, to decrease the number of possible identifications [96,97]. When no other approach can be used to ascertain skeletal remains of a human, the facial reconstruction technique - which is more of an identification device than others - may possibly be employed as a last resort or to supplement other primary methods [98-101]. The repeatability (test-retest reliability) and validation (accuracy) of measurements are two benefits of using the new CBCT [102-104]. The study of facial soft tissue and skull morphology forms the basis of this forensic tool, and CBCT provides information on both soft and hard tissues [105]. The location of landmarks is crucial for determining soft tissue thickness (STT), and it is believed that some of the discrepancies previously observed in the literature were caused by unclear guidelines regarding the definitions of these landmarks [106]. Katsumura et al. (2016) evaluated 47 landmarks, some of which were located on the mandibular and maxillary bones. They compared the real skull with a 3D model of the face created using CBCT [107]. They found that CBCT is a highly reproducible tool for dental use or the study of small spaces, for example, the oral cavity, because it can replicate measures like those of actual skulls. The results may be somewhat impacted by the presence of metal artifacts (p = 0.005 versus p = 0.011) [107].

Forensic identification frequently requires estimating the appearance of facial soft tissues from human skeletal remains. This procedure, a subfield of forensic facial anthropology, is also known as facial reconstruction or facial approximation [94,108,109]. In the 19th century, European painters used average soft tissue depths calculated from cadavers to shape clay over models of skulls, thereby approximating faces [94,110]. To precisely and impartially characterize the relationship between a skull and the soft tissue that covers it, CBCT has been employed in various computerized approaches over the past 20 years [111,112]. The complexity of the human skull is sufficiently distinct to be comparable to that of a fingerprint [113,114]. Claes et al. (2010) reported an extensive analysis that compiles recent developments in the field of computerized facial reconstruction into a single framework [115]. The following components comprise this framework: skull digitization, craniofacial model, target skull representation, model-to-skull registration, visualization, validation, and anthropological examination [115]. CBCT plays a crucial role in this procedure by facilitating the digitization of unknown skulls and the formation of craniofacial models [108,116].

Anthropometry offers a reliable method for assessing facial form and detecting variations in shape over time. This is crucial for the diagnosis of acquired anomalies, the planning and assessment of surgery, the investigation of normal and aberrant growth, and the distinction between normal growth and treatment outcomes [117]. These methods may enable the computation of angles, surface arcs, surface areas, and volumes of the face in addition to linear distances [118]. By decreasing the time required for tests and increasing measurement accuracy, image-processing techniques applied to facial images have the potential to enhance anthropometric applications. Automated measurement of therapeutically significant data would be possible with the extraction of desirable facial characteristics or landmarks [117]. Numerous tools can image the skull in a wide field of view, including most of the anthropometric landmarks used in cephalometric analysis. When measuring facial structures, maxillofacial CBCT has been shown to have high dimensional accuracy [119-121].

Craniofacial Indicators for Ancestry Determination

Forensic identification relies on estimating ancestry and ethnicity. There is limited information available regarding the use of CBCT in ancestry estimation; no research employing CBCT images of jaw-related cranial features or dental characteristics for ethnic estimation purposes was found. However, as it is an archaeological case, a study should be cited, even if it was not located using the search terms [122-124]. In this instance, the rASUDAS tool was utilized to score dental characteristics associated with ancestry by assessing root morphology using CBCT. This contemporary technical method, applied to a 1,500-year-old skull, may also apply to contemporary forensic cases, where the material is embrittled or challenging to access [122-124]. Further research is needed on this topic.

The impact of heritage on other biological précis factors, especially those related to age estimation, is another significant problem; if suitable models are not applied for those populations, erroneous estimations will occur [125-127]. For example, Du et al. (2021) and other studies have demonstrated that, on average, Chinese people have a greater pulp chamber volume than Black Americans [128]. Ancestry was not taken into consideration in many of the papers that were found for this evaluation [129]. Many did not identify the ethnic group, or, when they did, they disregarded subgroups of a larger ethnic cluster, such as those among Caucasians, the ethnicity of the ancestors of the individuals under study, or ethnicity in highly heterogeneous populations (like Afro-Americans, White, or Native Americans in Brazil) [130,131]. The percentages of the populations on which the investigations included in this paper were conducted. On the other hand, some scientists think that, at least when estimating age, using the correct statistical method is likely more significant than belonging to a particular ethnic group [132,133].

Dental Implant Backtracking for Human Identification

In the modern era of dental management, dental implants have become critical forensic evidence for human identification due to their unique designs, which include perforations, grooves, top chambers, and threads that only show up at specific rotations or angulations. Gopal (2018) conducted a panoramic radiography study for implant backtracking, which demonstrated an accuracy of 82.5%, opening the door for CBCT studies on the same topic for individual identification [134].

Determination of Facial Soft Tissue Thickness (FSTT) and Reconstructive Identification

When no AM records or presumptive identification are available, reconstructive identification is taken into consideration, particularly when the bones have been burned or macerated beyond recognition. It includes the coordinated use of 3D imaging (CT, CBCT, and MRI), anthropology, anatomy, forensic dentistry, and forensic medicine [33]. Both manual and computer-aided methods for reconstructive identification are used to restore the missing soft tissue, utilizing soft tissue pegs positioned at locations on the face that correspond to the average FSTT [94,135-137]. The computerized approach utilizes CT scanning or a laser video camera connected to a computer. Using this technique, the skull data can be visualized as a fully shaded 3D surface, allowing computer software to be used for facial design [138-140]. According to Farman and Scarfe (2006), CBCT image evaluation provides sufficient soft tissue definition and clarity to help identify the patient's profile and the air-soft tissue boundaries [141]. Fourie et al. (2010) compared the soft tissue - e.g., flesh, muscle, tendon, skin, fat, connective tissue, and ligament - thickness at 11 cephalometric points in cadaver heads using a dermal biopsy punch and CBCT with resolutions of 0.3 and 0.4 mm to assess the accuracy and dependability of CBCT scans in determining soft tissue diameter or heaviness of the face [142]. The dependability of CBCT for measuring FSTT with 0.3 mm resolution was established by their extremely high inter- and intra-observer correlation between the two techniques [141,142].

Evaluation and Demonstration of Cranial Trauma and Projectile Injuries

Imaging techniques, particularly postmortem computed tomography (PMCT), have been employed to investigate the fracture patterns in human calvarial injuries resulting from blunt force [143-145]. If a fictitious weapon is discovered, it can be compared to the impressed wounds on a person's skull. Because of the various bullet routes that may not be in the body or may be deflected by an anatomical structure, cases of firearm projectile injuries are typically challenging to evaluate [145-147]. Thus, being aware of the firearm projectile's location before the autopsy could help with the forensic analysis [148-150]. Stuehmer et al. (2009) revealed that CT remains the method of choice for imaging projectiles [151]. It is feasible to identify the most likely weapon because each combination of projectiles from a firearm is linked to a typical pattern of injuries.

Nevertheless, high-density projectiles result in significant CT artifacts, which make it challenging to study the anatomical structures near the projectile [150]. von See et al. (2009) examined tissue damage and the positions of three distinct contemporary projectiles that were fired into pig cadavers' skulls using CBCT and CT [152]. They concluded that because metallic artifacts in CT images have a less adverse impact on CBCT, it is considerably more effective at identifying structural osseous-tissue impairment in the immediate region of high-density projectiles. Stuehmer et al. (2009), and other studies that used CT and CBCT to examine viscerocranium gunshot injuries in 14 patients came to similar conclusions [150-155].

Unknown Skull Digitization

Initially, laser scanning technology was used to create a digital copy of the unidentified skull for loading into computer systems [156]. Thanks to advancements in medical imaging, CT is now a practical method for creating digital models of the skull. Today, CT scanners are used in all computerized reconstruction methods to digitize the skull [114]. Dental professionals frequently utilize CBCT, a type of medical CT that can digitize the skull with comparable resolution and lower radiation levels [157].

**Figure 3:**
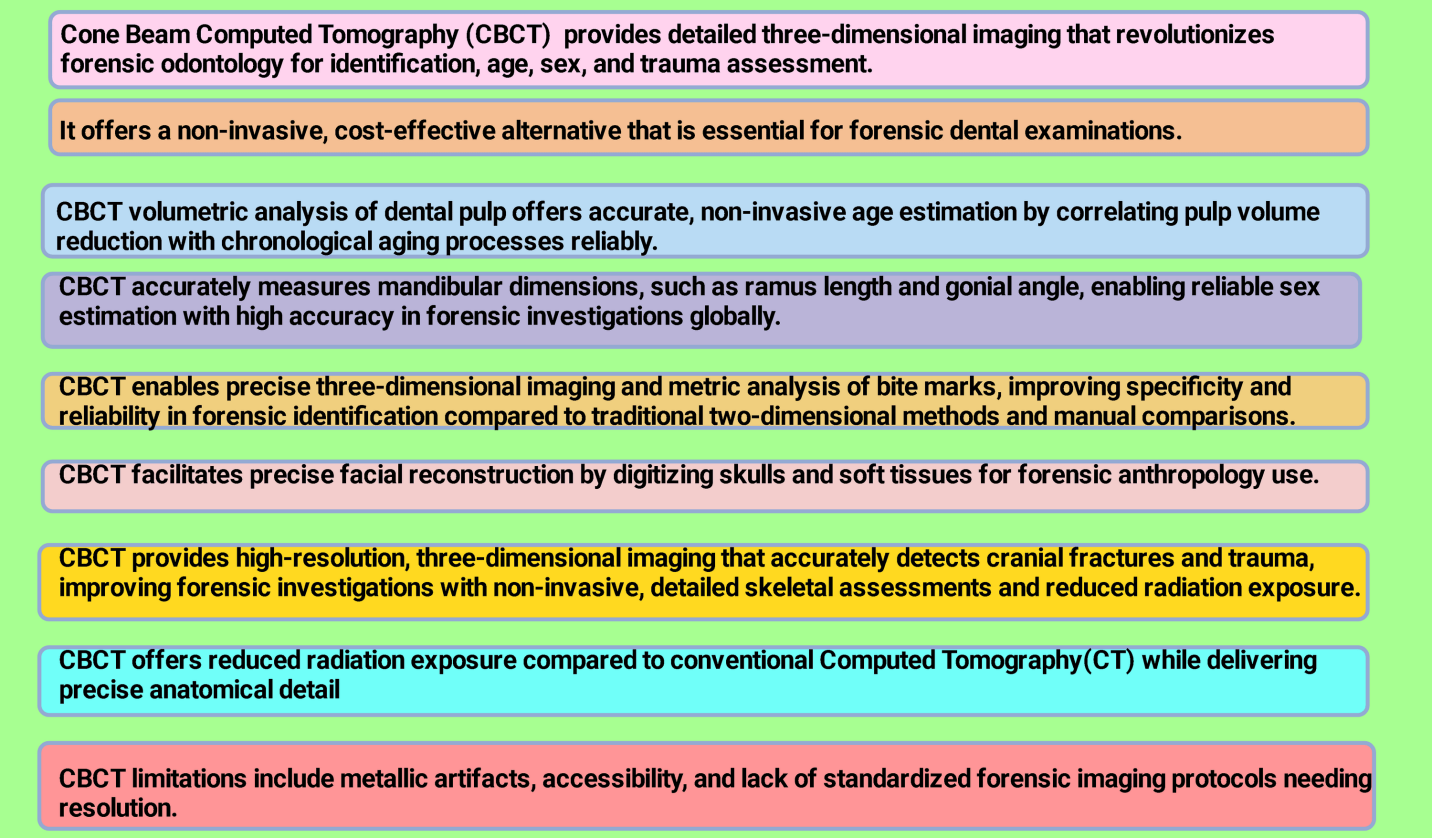
Key findings of this narrative review Note: This figure has been drawn utilizing the premium version of BioRender (https://BioRender.com/p2q6o8b) [41], accessed September 23rd, 2025, with agreement license number GR28SGAI1M. Illustration Credit: Rahnuma Ahmad

These days, investigations on facial reconstruction also include CBCT imaging. Every imaging method has drawbacks. Dental amalgam fillings introduce a considerable number of artifacts when scanned by CT and CBCT, whereas laser scanning procedures are limited by resolution and detail. One advantage of CBCT over CT is that it can provide images while the subject is seated, allowing for more comfortable imaging. The supine position of the patient during CT scanner operations may cause soft tissues to distort because of gravity [158]. The principal findings of this narrative review are depicted in Figure 3.

Limitations of CBCT Scientific Know-How

**Figure 4:**
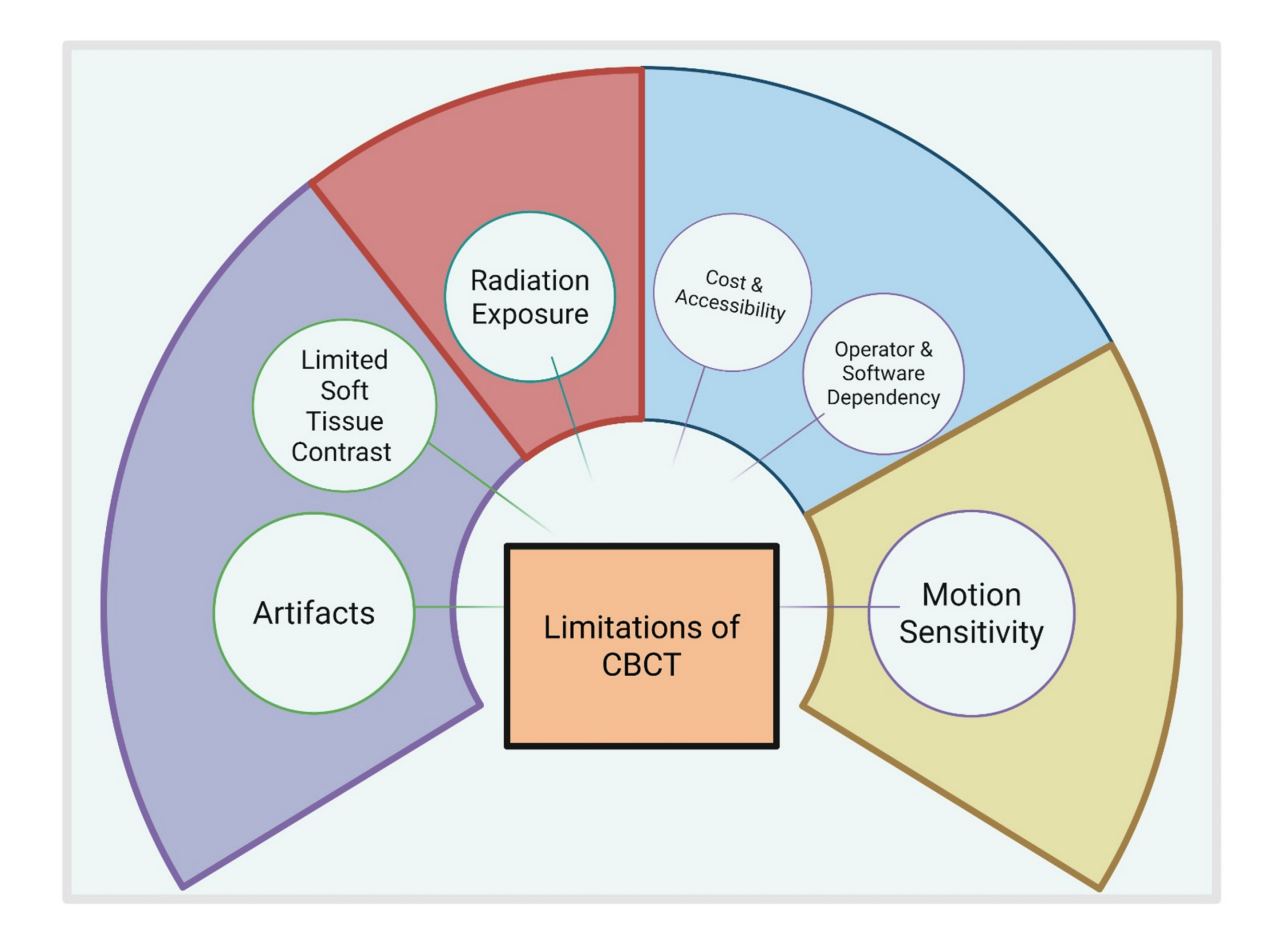
A flow chart illustrating the limitations of CBCT Note: This figure has been drawn utilizing the premium version of BioRender (https://BioRender.com/b3bpb5t) [41], accessed September 15th, 2025, with agreement license number JT28R7E1XV. Illustration Credit: Vishnu Desai

The most reported drawbacks of CBCT are the appearance of man-made objects in areas above and below the plane of rotation of the X-ray source, particularly when metallic elements are present, such as those found in crowns or restorations, during the reconstruction of 3D images [16]. Discrepancies between the actual content of the object being studied and its visible structure are known as artifacts. As a result, the diagnostic quality of the pictures declines [159]. It possibly bothers us as all this data and evidence are helpful for forensic identification when compared to AM data, even if the comparison process is not based just on it. It also impacts maxillofacial region analysis and limits diagnosis (Figure 4) [160].

Future research perspectives

Future research in the forensic applications of CBCT should focus on developing standardized imaging protocols explicitly tailored for forensic scenarios [123,124], particularly those involving mass disasters and unidentified remains. Advancements in artificial intelligence and machine learning offer promising avenues for enhancing the accuracy of CBCT-based identification, such as automating age and sex estimation through morphometric analysis. There is also a growing need to create population-specific CBCT reference databases, particularly for underrepresented ethnic groups, to enhance the reliability of forensic assessments. Furthermore, innovation in portable, field-ready CBCT devices could substantially improve the efficiency of forensic investigations in remote or disaster-affected areas.

Limitations of this review article

This review, being narrative in nature, is limited by the lack of quantitative synthesis or meta-analytical comparison. It relies on published literature, which may not uniformly represent all global populations or standardize CBCT techniques across studies. Furthermore, the article does not include original experimental data or clinical validation, and some of the discussed applications are still in early stages of research with limited practical implementation. The generalizability of conclusions is also constrained by variations in CBCT device settings, operator expertise, and software used for analysis.

Principal outcome of the narrative review

**Figure 5:**
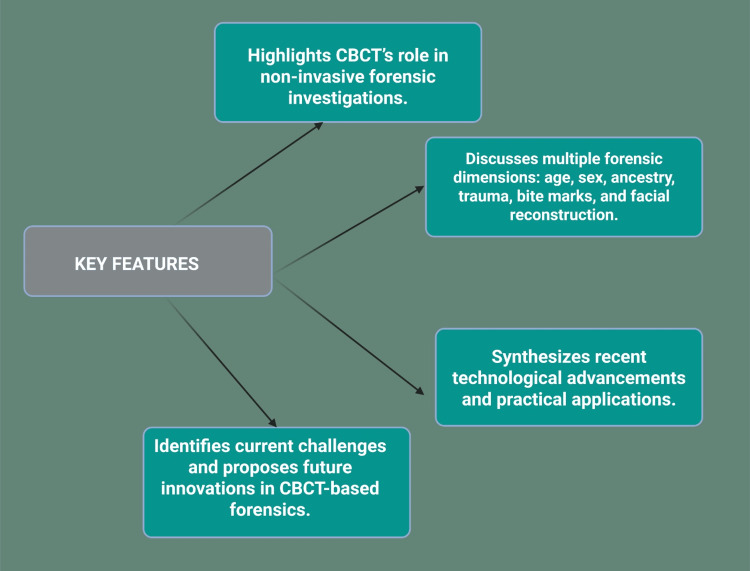
A flow chart illustrating the principal outcome of this paper Note: This figure was drawn using the premium version of BioRender (https://BioRender.com/p2q6o8b) [41], accessed June 27th, 2025, with an agreement license number KG28FU97WS. Illustration Credit: Vishnu Desai

This review highlights the unique role of CBCT in forensic science, emphasizing its non-invasive, high-resolution, and 3D imaging capabilities. It comprehensively covers its utility in various forensic domains, including personal identification, age, and sex estimation, bite mark analysis, ancestry profiling, facial reconstruction, and trauma evaluation. The article also highlights the integration of CBCT with digital software tools, which enhances visualization and diagnostic accuracy. By summarizing the strengths and challenges of CBCT in forensics, this review provides a valuable reference for researchers, forensic experts, and clinicians seeking to adopt advanced imaging in medico-legal investigations (Figure 5).

## Conclusions

CBCT has emerged as an indispensable tool in forensic odontology, offering unparalleled advantages in accuracy, visualization, and data preservation. Its applications range from basic identification to advanced reconstructive modeling. However, limitations regarding accessibility, artifacts, and lack of standardization must be addressed. With ongoing technological evolution and interdisciplinary collaboration, CBCT will continue to transform the landscape of forensic science.

## References

[1] (2025). American Academy of Forensic Sciences. Odontology.

[2] Gamba TO, Yamasaki MC, Groppo FC, da Silveira HL, Boscolo SM, Sanderink GC, Berkhout WE (2017). Validation study of a new method for sexual prediction based on CBCT analysis of maxillary sinus and mandibular canal. Arch Oral Biol.

[3] Gamba Tde O, Alves MC, Haiter-Neto F (2016). Mandibular sexual dimorphism analysis in CBCT scans. J Forensic Leg Med.

[4] Khandelwal S, Srivastava R, Mani Tripathi R, Dave M, Jain A, Shrivastav S (2025). Encoded in titanium: the digital DNA of forensic dentistry. Cureus.

[5] Khandelwal S, Srivastava R, Dave M, Jain A, Bais K, Shrivastav S (2025). Role of artificial intelligence in forensic odontology: a review. J Neonatal Surg.

[6] Asif MK, Nambiar P, Ibrahim N, Al-Amery SM, Khan IM (2019). Three-dimensional image analysis of developing mandibular third molars apices for age estimation: a study using CBCT data enhanced with Mimics & 3-Matics software. Leg Med (Tokyo).

[7] Porto LV, Celestino da Silva Neto J, Anjos Pontual AD, Catunda RQ (2015). Evaluation of volumetric changes of teeth in a Brazilian population by using cone beam computed tomography. J Forensic Leg Med.

[8] Esmaeilyfard R, Paknahad M, Dokohaki S (2021). Sex classification of first molar teeth in cone beam computed tomography images using data mining. Forensic Sci Int.

[9] Gulsahi A, Kulah CK, Bakirarar B, Gulen O, Kamburoglu K (2018). Age estimation based on pulp/tooth volume ratio measured on cone-beam CT images. Dentomaxillofac Radiol.

[10] Asif MK, Nambiar P, Mani SA, Ibrahim NB, Khan IM, Lokman NB (2019). Dental age estimation in Malaysian adults based on volumetric analysis of pulp/tooth ratio using CBCT data. Leg Med (Tokyo).

[11] Wanzeler AM, Alves-Júnior SM, Ayres L, da Costa Prestes MC, Gomes JT, Tuji FM (2019). Sex estimation using paranasal sinus discriminant analysis: a new approach via cone beam computerized tomography volume analysis. Int J Legal Med.

[12] Marroquin Penaloza TY, Karkhanis S, Kvaal SI (2016). Application of the Kvaal method for adult dental age estimation using cone beam computed tomography (CBCT). J Forensic Leg Med.

[13] Nemsi H, Haj Salem N, Bouanene I (2017). Age assessment in canine and premolar by cervical axial sections of cone-beam computed tomography. Leg Med (Tokyo).

[14] Pinchi V, Pradella F, Buti J, Baldinotti C, Focardi M, Norelli GA (2015). A new age estimation procedure based on the 3D CBCT study of the pulp cavity and hard tissues of the teeth for forensic purposes: a pilot study. J Forensic Leg Med.

[15] Mowafey B, Van de Casteele E, Youssef JM, Zaher AR, Omar H, Politis C, Jacobs R (2015). Can mandibular lingual canals be used as a forensic fingerprint?. J Forensic Odontostomatol.

[16] Eliášová H, Dostálová T (2017). 3D multislice and cone-beam computed tomography systems for dental identification. Prague Med Rep.

[17] Hajeer MY, Al-Homsi HK, Alfailany DT, Murad RM (2022). Evaluation of the diagnostic accuracy of CBCT-based interpretations of maxillary impacted canines compared to those of conventional radiography: an in vitro study. Int Orthod.

[18] Asif MK, Ibrahim N, Al-Amery SM, John J, Nambiar P (2019). Juvenile versus adult: a new approach for age estimation from 3-dimensional analyses of the mandibular third molar apices. J Forensic Radiol Imaging.

[19] Uğur Aydın Z, Bayrak S (2019). Relationship between pulp tooth area ratio and chronological age using cone-beam computed tomography images. J Forensic Sci.

[20] Trochesset DA, Serchuk RB, Colosi DC (2014). Generation of intra-oral-like images from cone beam computed tomography volumes for dental forensic image comparison. J Forensic Sci.

[21] Bayrak S, Halıcıoglu S, Kose G, Halıcıoglu K (2018). Evaluation of the relationship between mandibular condyle cortication and chronologic age with cone beam computed tomography. J Forensic Leg Med.

[22] Helmy MA, Osama M, Elhindawy MM, Mowafey B, Taalab YM, ElRahman HA (2020). Volume analysis of second molar pulp chamber using cone beam computed tomography for age estimation in Egyptian adults. J Forensic Odontostomatol.

[23] Nguyen E, Doyle E (2018). Dental post-mortem computed tomography for disaster victim identification: a literature review. J Forensic Radiol Imaging.

[24] Izham A, Auerkari EI (2021). The use of radiology CBCT in odontology forensic. AIP Conf Proc.

[25] Chandrasekhar T, Vennila P (2011). Role of radiology in forensic dentistry. J Indian Acad Oral Med Radiol.

[26] Scarfe WC, Farman AG, Sukovic P (2006). Clinical applications of cone-beam computed tomography in dental practice. J Can Dent Assoc.

[27] Tarani S, Kamakshi SS, Naik V, Sodhi A (2017). Forensic radiology: an emerging science. J Adv Clin Res Insights.

[28] Jain A, Shil M, Sreepradha C, Rai S, Kaur I, Banka A (2024). A review on cone-beam computed tomography and its application in dentistry. J Pharm Bioallied Sci.

[29] Sarment DP, Christensen AM (2014). The use of cone beam computed tomography in forensic radiology. J Forensic Radiol Imaging.

[30] Yang F, Jacobs R, Willems G (2006). Dental age estimation through volume matching of teeth imaged by cone-beam CT. Forensic Sci Int.

[31] Erickson M, Caruso JM, Leggitt L (2003). Newtom QR-DVT 9000 imagine used to confirm a clinical diagnosis of iatrogenic mandibular nerve paresthesia. J Calif Dent Assoc.

[32] Marques JAM, Musse JDO, Gois BC, Galvão LCC, Paranhos LR (2014). Cone-beam computed tomography analysis of the frontal sinus in forensic investigation. Int J Morphol.

[33] Issrani R, Prabhu N, Sghaireen MG (2022). Cone-beam computed tomography: a new tool on the horizon for forensic dentistry. Int J Environ Res Public Health.

[34] Chandran N, Gopal SK, Lankupalli AS (2024). Significance & correlation of paranasal sinuses volume and foramen magnum in forensics: a cone beam computed tomography study. J Indian Acad Forensic Med.

[35] Eckert WG, Garland N (1984). The history of the forensic applications in radiology. Am J Forensic Med Pathol.

[36] Chiam SL (2014). A note on digital dental radiography in forensic odontology. J Forensic Dent Sci.

[37] Eliasova H, Dostalova T, Prochazka A, Sediva E, Horacek M, Urbanova P, Hlinakova P (2021). Comparison of 2D OPG image versus orthopantomogram from 3D CBCT from the forensic point of view. Leg Med (Tokyo).

[38] Rubin GD (2014). Computed tomography: revolutionizing the practice of medicine for 40 years. Radiology.

[39] Ai T, Morelli JN, Hu X, Hao D, Goerner FL, Ager B, Runge VM (2012). A historical overview of magnetic resonance imaging, focusing on technological innovations. Invest Radiol.

[40] Rocha Sdos S, Ramos DL, Cavalcanti Mde G (2003). Applicability of 3D-CT facial reconstruction for forensic individual identification. Pesqui Odontol Bras.

[41] (2025). Biorender. https://www.biorender.com/.

[42] Venkatesh E, Elluru SV (2017). Cone beam computed tomography: basics and applications in dentistry. J Istanb Univ Fac Dent.

[43] Asif MK, Nambiar P, Khan IM, Aziz ZA, Noor NS, Shanmuhasuntharam P, Ibrahim N (2019). Enhancing the three-dimensional visualization of a foreign object using Mimics software. Radiol Case Rep.

[44] Wang J, Huang Z, Wang F, Yu X, Li D (2020). Materialise's interactive medical image control system (MIMICS) is feasible for volumetric measurement of urinary calculus. Urolithiasis.

[45] Gohain M, Asif MK, Nambiar P, Mohd Noor NS, Hidayah Reduwan N, Ibrahim N (2024). Three-dimensional surface area analyses of developing maxillary second premolar root apices for age estimation using CBCT images. Leg Med (Tokyo).

[46] Kumar R, Athota A, Rastogi T, Karumuri S (2015). Forensic radiology: an emerging tool in identification. J Indian Acad Oral Med Radiol.

[47] Shaibah WI, Yamany IA, Jastaniah SD (2014). Physical measurements for the accuracy of cone-beam CT in dental radiography. Open J Med Imaging.

[48] Wu Y, Chen X, Shen Y (2013). Effectiveness assessment of 3-D cone beam CT used in human bite marks identification (Article in Chinese). Sheng Wu Yi Xue Gong Cheng Xue Za Zhi.

[49] Marques J, Musse J, Caetano C, Corte-Real F, Corte-Real AT (2013). Analysis of bite marks in foodstuffs by computer tomography (cone beam CT) - 3D reconstruction. J Forensic Odontostomatol.

[50] Martin-de las Heras S, Valenzuela A, Javier Valverde A, Torres JC, Luna-del-Castillo JD (2007). Effectiveness of comparison overlays generated with DentalPrint software in bite mark analysis. J Forensic Sci.

[51] Blackwell SA, Taylor RV, Gordon I (2007). 3-D imaging and quantitative comparison of human dentitions and simulated bite marks. Int J Legal Med.

[52] Daniel MJ, Pazhani A (2015). Accuracy of bite mark analysis from food substances: a comparative study. J Forensic Dent Sci.

[53] Naether S, Buck U, Campana L, Breitbeck R, Thali M (2012). The examination and identification of bite marks in foods using 3D scanning and 3D comparison methods. Int J Legal Med.

[54] Star H, Thevissen P, Jacobs R, Fieuws S, Solheim T, Willems G (2011). Human dental age estimation by calculation of pulp-tooth volume ratios yielded on clinically acquired cone beam computed tomography images of monoradicular teeth. J Forensic Sci.

[55] Archer MS, Jones SD, Wallman JF (2018). Delayed reception of live blowfly (Calliphora vicina and Chrysomya rufifacies) larval samples: implications for minimum postmortem interval estimates. Forensic Sci Res.

[56] Davies J, Johnson B, Drage N (2012). Effective doses from cone beam CT investigation of the jaws. Dentomaxillofac Radiol.

[57] Giri S, Tripathi A, Patil R, Khanna V, Singh V (2019). Analysis of bite marks in food stuffs by CBCT 3D-reconstruction. J Oral Biol Craniofac Res.

[58] Ali IK, Sansare K, Karjodkar FR (2018). Analysis of intercanine distance and dimensional changes in bite marks on foodstuffs using cone beam computed tomography. Am J Forensic Med Pathol.

[59] Koh KK, Tan JS, Nambiar P, Ibrahim N, Mutalik S, Khan Asif M (2017). Age estimation from structural changes of teeth and buccal alveolar bone level. J Forensic Leg Med.

[60] Pires AC, de Sousa Santos RF, Pereira CP (2021). Dental age assessment by the pulp/tooth area proportion in cone beam computed tomography: is medico-legal application for age estimation reliable?. J Forensic Odontostomatol.

[61] Zhang ZY, Yan CX, Min QM (2019). Age estimation using pulp/enamel volume ratio of impacted mandibular third molars measured on CBCT images in a northern Chinese population. Int J Legal Med.

[62] Molina A, Bravo M, Fonseca GM, Márquez-Grant N, Martín-de-Las-Heras S (2021). Dental age estimation based on pulp chamber/crown volume ratio measured on CBCT images in a Spanish population. Int J Legal Med.

[63] Merdietio Boedi R, Shepherd S, Mânica S, Franco A (2022). CBCT in dental age estimation: a systematic review and meta analysis. Dentomaxillofac Radiol.

[64] Yüksel İB, Altındağ A (2025). Age estimation based on pulp/tooth volume ratio using CBCT: a study on mandibular canines. BMC Oral Health.

[65] Marques-Moura S, Caldas IM (2024). Study of secondary dentine deposition in central incisors as an age estimation method for adults. Forensic Sci Med Pathol.

[66] Kazmi S, Mânica S, Revie G, Shepherd S, Hector M (2019). Age estimation using canine pulp volumes in adults: a CBCT image analysis. Int J Legal Med.

[67] Hidayat SR, Oscandar F, Malinda Y, Lita YA (2018). Human age estimation based on pulp volume of canines for chronological age estimation: preliminary research. Padjadjaran J Dent.

[68] Salvatierra JM, Díaz-Suyo A, Alvarado ER, Kapoor P, Chowdhry A, Velezmoro YW, Rivas AG (2023). Dental age estimation by assessing the pulp volume of five teeth in a Peruvian population using cone beam computed tomography images. ODOVTOS Int J Dental Sci.

[69] Safaei A, Bagherpour A, Naseri S, Etemadi M, Khoshkhou H (2024). Age estimation by evaluation of pulp chamber to crown volume of central incisor and first molar of maxilla, using cone-beam CT. Heliyon.

[70] Hassanaly M, Morais Caldas I, Teixeira A, Pérez-Mongiovi D (2023). Application of CBCT technology in forensic odontology: a narrative review. Curr Forensic Sci.

[71] Shahin KA, Chatra L, Shenai P (2013). Dental and craniofacial imaging in forensics. J Forensic Radiol Imaging.

[72] Santos MA, Muinelo-Lorenzo J, Fernández-Alonso A, Cruz-Landeira A, Aroso C, Suárez-Cunqueiro MM (2022). Age estimation using maxillary central incisor analysis on cone beam computed tomography human images. Int J Environ Res Public Health.

[73] Demirjian A, Goldstein H (1976). New systems for dental maturity based on seven and four teeth. Ann Hum Biol.

[74] Chaurasia A, Giri S, Katheriya G, Patil R (2016). CBCT in forensic odontology. Indian J Forensic Odontol.

[75] Capitaneanu C, Willems G, Thevissen P (2017). A systematic review of odontological sex estimation methods. J Forensic Odontostomatol.

[76] Rivera-Mendoza F, Martín-de-Las-Heras S, Navarro-Cáceres P, Fonseca GM (2018). Bite mark analysis in foodstuffs and inanimate objects and the underlying proofs for validity and judicial acceptance. J Forensic Sci.

[77] Paknahad M, Dokohaki S, Khojastepour L, Shahidi S, Haghnegahdar A (2022). A radio-odontometric analysis of sexual dimorphism in first molars using cone-beam computed tomography. Am J Forensic Med Pathol.

[78] Manhaes-Caldas D, Oliveira ML, Groppo FC, Haiter-Neto F (2019). Volumetric assessment of the dental crown for sex estimation by means of cone-beam computed tomography. Forensic Sci Int.

[79] Lin E, Alessio A (2009). What are the basic concepts of temporal, contrast, and spatial resolution in cardiac CT?. J Cardiovasc Comput Tomogr.

[80] Sahithi D, Reddy S, Divya Teja DV, Koneru J, Sai Praveen KN, Sruthi R (2016). Reveal the concealed - morphological variations of the coronoid process, condyle, and sigmoid notch in personal identification. Egypt J Forensic Sci.

[81] Kharoshah MA, Almadani O, Ghaleb SS, Zaki MK, Fattah YA (2010). Sexual dimorphism of the mandible in a modern Egyptian population. J Forensic Leg Med.

[82] Okkesim A, Sezen Erhamza T (2020). Assessment of mandibular ramus for sex determination: retrospective study. J Oral Biol Craniofac Res.

[83] Munhoz L, Okada S, Hisatomi M, Yanagi Y, Arita ES, Asaumi J (2024). Are computed tomography images of the mandible useful in age and sex determination? A forensic science meta-analysis. J Forensic Odontostomatol.

[84] Rhee CH, Shin SM, Choi YS (2015). Application of statistical shape analysis for the estimation of bone and forensic age using the shapes of the 2nd, 3rd, and 4th cervical vertebrae in a young Japanese population. Forensic Sci Int.

[85] Costa ED, Peyneau PD, Roque-Torres GD, Freitas DQ, Ramírez-Sotelo LR, Ambrosano GM, Verner FS (2019). The relationship of articular eminence and mandibular fossa morphology to facial profile and gender determined by cone beam computed tomography. Oral Surg Oral Med Oral Pathol Oral Radiol.

[86] Toneva DH, Nikolova SY, Fileva NF, Zlatareva DK (2023). Size and shape of human mandible: sex differences and influence of age on sex estimation accuracy. Leg Med (Tokyo).

[87] Taleb NSA, Beshlawy ME (2015). Mandibular ramus and gonial angle measurements as predictors of sex and age in an Egyptian population sample: a digital panoramic study. J Forensic Res.

[88] Leversha J, McKeough G, Myrteza A, Skjellrup-Wakefiled H, Welsh J, Sholapurkar A (2016). Age and gender correlation of gonial angle, ramus height and bigonial width in dentate subjects in a dental school in Far North Queensland. J Clin Exp Dent.

[89] Damera A, Mohanalakhsmi J, Yellarthi PK, Rezwana BM (2016). Radiographic evaluation of mandibular ramus for gender estimation: retrospective study. J Forensic Dent Sci.

[90] Arthanari A, Ravindran V, Ramalingam K, Prathap L, Raj S (2024). Gender determination through mandibular features on orthopantomograms: a preliminary study. Cureus.

[91] Steyn M, Işcan MY (1998). Sexual dimorphism in the crania and mandibles of South African Whites. Forensic Sci Int.

[92] Saini V, Srivastava R, Shamal SN, Singh TB, Pandey AK, Tripathi SK (2011). Sex determination using mandibular ramus flexure: a preliminary study on Indian population. J Forensic Leg Med.

[93] Arthanari A, Sureshbabu S, Ramalingam K, Ravindran V, Prathap L (2024). Quantifying sexual dimorphism by analyzing ramus flexure and bigonial width in orthopantomography. Cureus.

[94] Gupta S, Gupta V, Vij H, Vij R, Tyagi N (2015). Forensic facial reconstruction: the final frontier. J Clin Diagn Res.

[95] Setiawardania A, Ur Rahman A, Utama AN, Prakosa JS, Susilo YA (2025). The role of artificial intelligence in the development of 3D facial reconstructions on skull bones as a forensic investigation solution. J Indonesia Sos Teknologi.

[96] Patel AS, Suneel AT, Singh J, Chitravanshi S (2023). Facial reconstruction: a boon to forensic practice. Int J Foren Med.

[97] Evison MP, Iwamura ESM, Guimarães MA, Schofield D (2016). Forensic facial reconstruction and its contribution to identification in missing person cases. Handbook of Missing Persons.

[98] Navic P, Inthasan C, Chaimongkhol T, Mahakkanukrauh P (2023). Facial reconstruction using 3-D computerized method: a scoping review of methods, current status, and future developments. Leg Med (Tokyo).

[99] Satelur KP, Annegowda VM, Thanushree N, Adavi S, Murthy SK, Megha S (2024). From bones to identity: the forensic sciences of facial reconstruction. Afr J Biomed Res.

[100] Dahal A, McNevin D, Chikhani M, Ward J (2023). An interdisciplinary forensic approach for human remains identification and missing persons investigations. WIREs Forensic Sci.

[101] Pilli E, Palamenghi A, Cattaneo C (2025). Forensic skeletal and molecular anthropology face to face: combining expertise for identification of human remains. Ann N Y Acad Sci.

[102] Ozdede M, Akay G, Karadag Atas O, Koc EK, Yalcin O, Gungor K (2024). Repeatability and effect of different voxel sizes on linear and volumetric tooth and pulp measurements using cone-beam computed tomography. BMC Oral Health.

[103] Patcas R, Markic G, Müller L, Ullrich O, Peltomäki T, Kellenberger CJ, Karlo CA (2012). Accuracy of linear intraoral measurements using cone beam CT and multidetector CT: a tale of two CTs. Dentomaxillofac Radiol.

[104] Ferreira JB, Christovam IO, Alencar DS, da Motta AF, Mattos CT, Cury-Saramago A (2017). Accuracy and reproducibility of dental measurements on tomographic digital models: a systematic review and meta-analysis. Dentomaxillofac Radiol.

[105] Hwang HS, Choe SY, Hwang JS, Moon DN, Hou Y, Lee WJ, Wilkinson C (2015). Reproducibility of facial soft tissue thickness measurements using cone-beam CT images according to the measurement methods. J Forensic Sci.

[106] Beaini TL, Miamoto P, Duailibi-Neto EF, Tedeschi-Oliveira SV, Chilvarquer I, Melani RF (2021). Facial soft tissue depth measurements in cone-beam computed tomography: a study of a Brazilian sample. Leg Med (Tokyo).

[107] Katsumura S, Sato K, Ikawa T, Yamamura K, Ando E, Shigeta Y, Ogawa T (2016). "High-precision, reconstructed 3D model" of skull scanned by conebeam CT: reproducibility verified using CAD/CAM data. Leg Med (Tokyo).

[108] Jayakrishnan JM, Kumar RBV (2022). Forensic facial reconstruction using CBCT: a systematic review. Stomatologija.

[109] Stoney MB, Koelmeyer TD (1999). Facial reconstruction: a case report and review of development of techniques. Med Sci Law.

[110] Lindsay KE, Rühli FJ, Deleon VB (2015). Revealing the face of an ancient Egyptian: synthesis of current and traditional approaches to evidence-based facial approximation. Anat Rec (Hoboken).

[111] Januário AL, Barriviera M, Duarte WR (2008). Soft tissue cone-beam computed tomography: a novel method for the measurement of gingival tissue and the dimensions of the dentogingival unit. J Esthet Restor Dent.

[112] Stratemann SA, Huang JC, Maki K, Miller AJ, Hatcher DC (2008). Comparison of cone beam computed tomography imaging with physical measures. Dentomaxillofac Radiol.

[113] Stampacchia S, Asadi S, Tomczyk S (2024). Fingerprints of brain disease: connectome identifiability in Alzheimer's disease. Commun Biol.

[114] Van De Ville D, Farouj Y, Preti MG, Liégeois R, Amico E (2021). When makes you unique: temporality of the human brain fingerprint. Sci Adv.

[115] Claes P, Vandermeulen D, De Greef S, Willems G, Clement JG, Suetens P (2010). Computerized craniofacial reconstruction: conceptual framework and review. Forensic Sci Int.

[116] Lee SY, Kim H, Lee D, Park C (2021). Superimposition of a cone beam computed tomography (CBCT) scan and a photograph: a dental technique. J Prosthet Dent.

[117] Douglas TS (2004). Image processing for craniofacial landmark identification and measurement: a review of photogrammetry and cephalometry. Comput Med Imaging Graph.

[118] Weinberg SM, Scott NM, Neiswanger K, Brandon CA, Marazita ML (2004). Digital three-dimensional photogrammetry: evaluation of anthropometric precision and accuracy using a Genex 3D camera system. Cleft Palate Craniofac J.

[119] Hilgers ML, Scarfe WC, Scheetz JP, Farman AG (2005). Accuracy of linear temporomandibular joint measurements with cone beam computed tomography and digital cephalometric radiography. Am J Orthod Dentofacial Orthop.

[120] Hu KS, Choi DY, Lee WJ, Kim HJ, Jung UW, Kim S (2012). Reliability of two different presurgical preparation methods for implant dentistry based on panoramic radiography and cone-beam computed tomography in cadavers. J Periodontal Implant Sci.

[121] Lascala CA, Panella J, Marques MM (2004). Analysis of the accuracy of linear measurements obtained by cone beam computed tomography (CBCT-NewTom). Dentomaxillofac Radiol.

[122] Štamfelj I, Hitij T, Leben-Seljak P (2019). Dental ancestry estimation in a 1500 years old human skeleton from Slovenia using a new web-based application rASUDAS. J Forensic Odontostomatol.

[123] Alhawasli RY, Ajaj MA, Hajeer MY, Al-Zahabi AM, Mahaini L (2022). Volumetric analysis of the jaws in skeletal class I and III patients with different facial divergence using CBCT imaging. Radiol Res Pract.

[124] Pasbola A, Puri A, Nangia R, Bhat S, Ahmed J, Nagar N (2024). Forensic dentistry’s invaluable contribution to human identification. Arch Dent Res.

[125] Liversidge HM, Peariasamy K, Folayan MO (2017). A radiographic study of the mandibular third molar root development in different ethnic groups. J Forensic Odontostomatol.

[126] Dhamo B, Kragt L, Grgic O (2018). Ancestry and dental development: a geographic and genetic perspective. Am J Phys Anthropol.

[127] Olze A, Schmeling A, Taniguchi M, Maeda H, van Niekerk P, Wernecke KD, Geserick G (2004). Forensic age estimation in living subjects: the ethnic factor in wisdom tooth mineralization. Int J Legal Med.

[128] Du H, Li G, Zheng Q, Yang J (2022). Population-specific age estimation in Black Americans and Chinese people based on pulp chamber volume of first molars from cone beam computed tomography. Int J Legal Med.

[129] Mauro M, Allen DS, Dauda B, Molina SJ, Neale BM, Lewis AC (2022). A scoping review of guidelines for the use of race, ethnicity, and ancestry reveals widespread consensus but also points of ongoing disagreement. Am J Hum Genet.

[130] Lu C, Ahmed R, Lamri A, Anand SS (2022). Use of race, ethnicity, and ancestry data in health research. PLOS Glob Public Health.

[131] Dauda B, Molina SJ, Allen DS (2023). Ancestry: how researchers use it and what they mean by it. Front Genet.

[132] Ubelaker DH, Khosrowshahi H (2019). Estimation of age in forensic anthropology: historical perspective and recent methodological advances. Forensic Sci Res.

[133] Willmann C, Fernandez De Grado G, Kolb C, Raul JS, Musset AM, Gros CI, Offner D (2023). Accuracy of age estimation using three dental age estimation methods in a young, large, and multiethnic patient sample. Dent J (Basel).

[134] Gopal SK (2018). Role of 3D cone beam computed tomography imaging in forensic dentistry: a review of the literature. Indian Forensic Odontol.

[135] Kreutz K, Verhoff MA (2007). Forensic facial reconstruction - identification based on skeletal findings. Dtsch Arztebl.

[136] Gietzen T, Brylka R, Achenbach J (2019). A method for automatic forensic facial reconstruction based on dense statistics of soft tissue thickness. PLoS One.

[137] Preuß S, Becker S, Rosenfelder J, Labudde D (2025). Computer-aided facial soft tissue reconstruction with computer vision: a modern approach to identifying unknown individuals. Appl Sci.

[138] Hildebolt CF, Vannier MW, Knapp RH (1990). Validation study of skull three-dimensional computerized tomography measurements. Am J Phys Anthropol.

[139] Vanezi P, Vanezis M, McCombe G, Niblett T (2000). Facial reconstruction using 3-D computer graphics. Forensic Sci Int.

[140] Giménez-El-Amrani A, Sanz-Garcia A, Villalba-Rojas N, Mirabet V, Valverde-Navarro A, Escobedo-Lucea C (2024). The untapped potential of 3D virtualization using high resolution scanner-based and photogrammetry technologies for bone bank digital modeling. Comput Biol Med.

[141] Farman AG, Scarfe WC (2006). Development of imaging selection criteria and procedures should precede cephalometric assessment with cone-beam computed tomography. Am J Orthod Dentofacial Orthop.

[142] Fourie Z, Damstra J, Gerrits PO, Ren Y (2010). Accuracy and reliability of facial soft tissue depth measurements using cone beam computer tomography. Forensic Sci Int.

[143] Wood RE (2006). Forensic aspects of maxillofacial radiology. Forensic Sci Int.

[144] Kranioti EF (2015). Forensic investigation of cranial injuries due to blunt force trauma: current best practice. Res Rep Forensic Med Sci.

[145] Kara-Boulad JM, Burhan AS, Hajeer MY, Nawaya FR, Jaber ST (2025). CBCT-based assessment of apical root resorption and alveolar bone height following orthodontic treatment of class I moderate crowding with labial vs. lingual fixed appliances in young adults: a randomized controlled trial. Int Orthod.

[146] de Bakker B, Soerdjbalie-Maikoe V, de Bakker HM (2013). The use of 3D-CT in weapon caused impression fractures of the skull, from a forensic radiological point of view. J Forensic Radiol Imaging.

[147] Eze UO, Ojifinni KA (2022). Trauma forensics in blunt and sharp force injuries. J West Afr Coll Surg.

[148] Kirchhoff SM, Scaparra EF, Grimm J, Scherr M, Graw M, Reiser MF, Peschel O (2016). Postmortem computed tomography (PMCT) and autopsy in deadly gunshot wounds--a comparative study. Int J Legal Med.

[149] Shrestha K, Shrestha N, Yadav S, Yadav D (2024). Localization of a bullet in a firearm injury victim using X-ray imaging during autopsy: a case report. JNMA J Nepal Med Assoc.

[150] Wankhade T, Sharma RK, Patil A, Rastogi AK, Goel N (2024). Where is the bullet? A puzzling case of an untraceable projectile in a gunshot injury. Cureus.

[151] Stuehmer C, Blum KS, Kokemueller H, Tavassol F, Bormann KH, Gellrich NC, Rücker M (2009). Influence of different types of guns, projectiles, and propellants on patterns of injury to the viscerocranium. J Oral Maxillofac Surg.

[152] von See C, Bormann KH, Schumann P, Goetz F, Gellrich NC, Rücker M (2009). Forensic imaging of projectiles using cone-beam computed tomography. Forensic Sci Int.

[153] Amole O, Osunde O, Akhiwu B, Efunkoya A, Omeje K, Amole T, Iliyasu Z (2017). A 14-year review of craniomaxillofacial gunshot wounds in a resource-limited setting. Craniomaxillofac Trauma Reconstr.

[154] Hanna TN, Shuaib W, Han T, Mehta A, Khosa F (2015). Firearms, bullets, and wound ballistics: an imaging primer. Injury.

[155] Akinniyi TA, Aregbesola SB, Famurewa BA, Akomolafe AG (2022). Civilian gunshot orofacial injury in a Nigerian tertiary hospital: a 10-year retrospective review. Ann Ib Postgrad Med.

[156] Moss JP, Linney AD, Grindrod SR, Mosse CA (1989). A laser scanning system for the measurement of facial surface morphology. Opt Lasers Eng.

[157] Mah JK, Yi L, Huang RC, Choo H (2011). Advanced applications of cone beam computed tomography in orthodontics. Semin Orthod.

[158] Vander Pluym J, Shan WW, Taher Z (2007). Use of magnetic resonance imaging to measure facial soft tissue depth. Cleft Palate Craniofac J.

[159] Mancini AX, Santos MU, Gaêta-Araujo H, Tirapelli C, Pauwels R, Oliveira-Santos C (2021). Artefacts at different distances from titanium and zirconia implants in cone-beam computed tomography: effect of tube current and metal artefact reduction. Clin Oral Investig.

[160] Pauwels R, Seynaeve L, Henriques JC (2015). Optimization of dental CBCT exposures through mAs reduction. Dentomaxillofac Radiol.

